# The role of major immune cells in myocardial infarction

**DOI:** 10.3389/fimmu.2022.1084460

**Published:** 2023-01-19

**Authors:** Qiang Feng, Qirong Li, Hengzong Zhou, Liqun Sun, Chao Lin, Ye Jin, Dongxu Wang, Gongliang Guo

**Affiliations:** ^1^ Department of Cardiology, China-Japan Union Hospital of Jilin University, Changchun, China; ^2^ Laboratory Animal Center, College of Animal Science, Jilin University, Changchun, China; ^3^ Department of Pathogenobiology, Jilin University Mycology Research Center, College of Basic Medical Sciences, Jilin University, Changchun, China; ^4^ School of Grain Science and Technology, Jilin Business and Technology College, Changchun, China; ^5^ School of Pharmacy, Changchun University of Chinese Medicine, Changchun, China

**Keywords:** myocardial infarction, immune cells, non-specific immunity, specific immunity, animal model, heart patch

## Abstract

Myocardial infarction (MI) is a cardiovascular disease (CVD) with high morbidity and mortality worldwide, often leading to adverse cardiac remodeling and heart failure, which is a serious threat to human life and health. The immune system makes an important contribution to the maintenance of normal cardiac function. In the disease process of MI, necrotic cardiomyocytes release signals that activate nonspecific immunity and trigger the action of specific immunity. Complex immune cells play an important role in all stages of MI progression by removing necrotic cardiomyocytes and tissue and promoting the healing of damaged tissue cells. With the development of biomaterials, cardiac patches have become an emerging method of repairing MI, and the development of engineered cardiac patches through the construction of multiple animal models of MI can help treat MI. This review introduces immune cells involved in the development of MI, summarizes the commonly used animal models of MI and the newly developed cardiac patch, so as to provide scientific reference for the accurate diagnosis and effective treatment of MI.

## 1 Introduction

Myocardial infarction (MI) is a major cardiovascular disease that may lead to death (1). MI is permanent damage to the myocardium caused by prolonged ischemia, which mainly occurs in the left ventricle, and will lead to diffuse discomfort from the chest to all parts of the body (2, 3). MI mainly includes spontaneous MI caused by primary coronary events, secondary MI caused by decreased oxygen supply or increased oxygen demand to the myocardium, and unexpected sudden cardiac death such as cardiac arrest (3). MI can be detected in several ways, the electrocardiogram shows the appearance of Q waves and dynamic changes in the ST-T segment waves, and damaged cardiomyocytes release substances such as myoglobin, lactate dehydrogenase, and creatine kinase (2).

The immune system is an inherent health defense system of animals, an interactive network of lymphoid organs, immune cells, and immunoreactive substances (4). The entire progression of the heart is regulated by the body’s immune system, which plays an important role in healing and remodeling after MI (5). Immune cells are the essential elements that support the immune system in its immune role. Immunity is divided into nonspecific and specific immunity (6). Nonspecific immunity is a natural immune defense developed during the long-term evolution of organisms, providing immediate and conservative host defense that may damage normal tissues because of its nonspecific nature (6). Specific immunity is a kind of acquired and precise immunity that mediates cellular and humoral immunity through T and B cells respectively (7).

After MI occurs, myocardial cells die and necrosis occurs in the tissue of the infarcted region, activating an inflammatory response. Immune cells can both promote cardiomyocyte death and inflammation, and facilitate the regeneration of damaged heart muscle (8). Animal model is an important part of disease research, and the construction of animal models that match the phenotype of human MI is essential for the in-depth study of the mechanism of immune cells and therapeutic approaches (9). The development of biological materials provides a new platform for the treatment of MI and is one of the important methods to achieve repair of the damaged heart (10). Engineered heart patches have been the focus of research in recent years for the development of materials that enable the damaged heart to self-renew (11). This review will be useful in elucidating the immune cells, animal models, and materials associated with MI.

## 2 Immune cell

**Figure 1:**
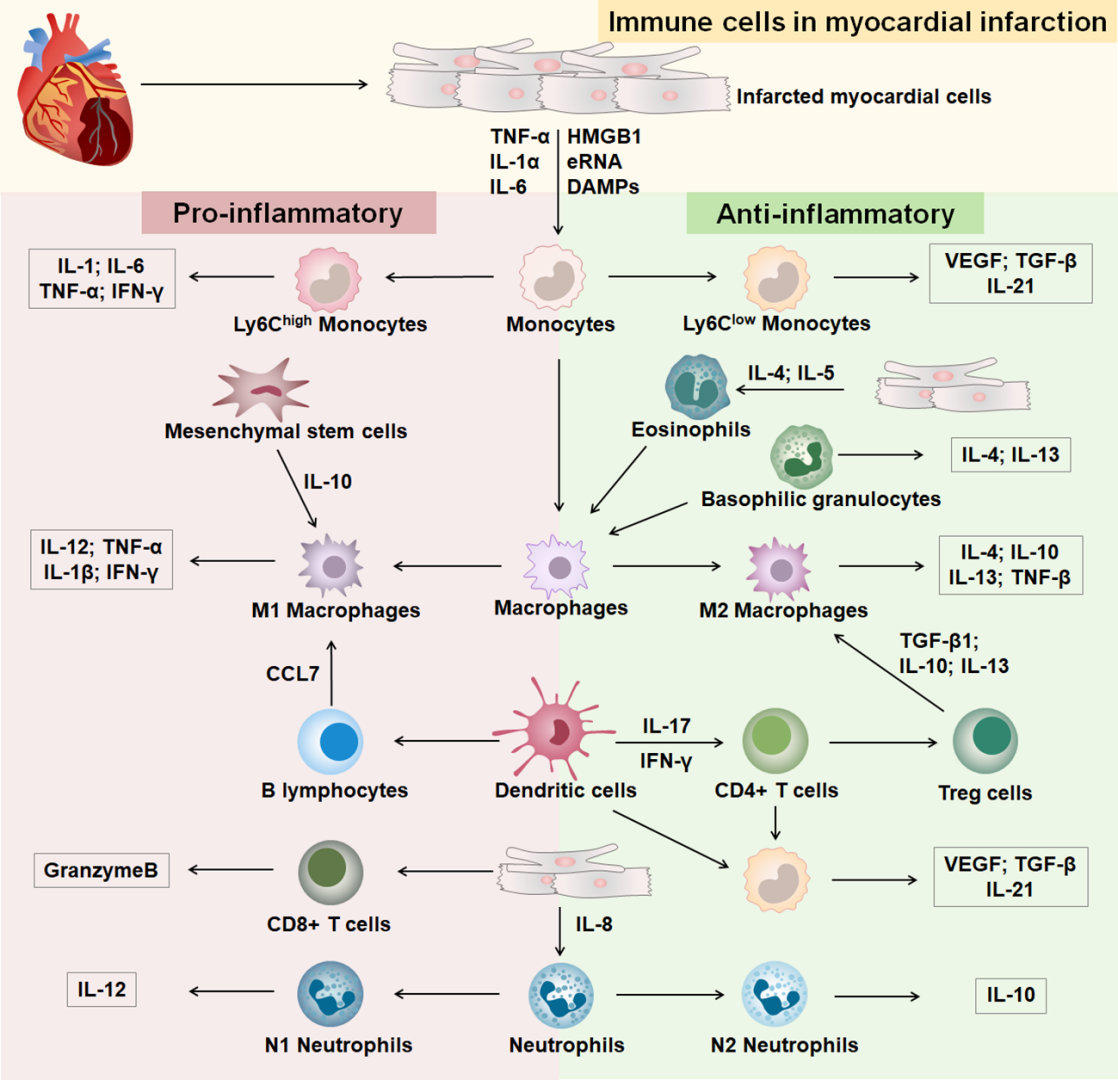
Immune cells in MI. The part with pink background on the left is immune cells differentiated to a pro-inflammatory phenotype after MI, and the part with green background on the right is immune cells differentiated to an anti-inflammatory phenotype after MI.

Heart healing after MI goes through three successive stages of development: inflammation, hyperplasia, and maturation (12) (**Supplementary Figure 1**). Different immune cells infiltrate at different stages of development in response to MI (**Figure 1**). In the early stages of MI, necrotic myocytes release damage-associated molecular patterns, cytokines, and autoantigens (13). After the onset of MI, an inflammatory outbreak first activates the immune response of nonspecific immune cells, causing rapid entry of monocytes, neutrophils, and dendritic cells from the peripheral vasculature into the infarcted area (14, 15). Subsequently, the infarcted myocardium continues to recruit macrophages that polarize into anti-inflammatory macrophages, which activate the repair of MI by secreting anti-inflammatory cytokines, promoting the formation of granulation tissue, and eliminating dead myocardial cells (16). After the inflammatory phase, a specific immune response initiates the repair of myocardial injury, the extracellular matrix begins to reconstitute, and T and B lymphocytes infiltrate the infarcted area in large numbers (17, 18). T cells are activated by dendritic cells, and regulatory T cells produce cytokines to induce macrophages to polarize and promote myocardial healing (13). Insight into immune cells in MI contributes to the identification of effective therapeutic targets.

### 2.1 Role of the mononuclear phagocytic system in MI

The mononuclear phagocytic system is generated by bone marrow progenitor cells and includes monocytes, macrophages, and dendritic cells (DCs) (19). Monocytes and macrophages are emerging therapeutic targets in cardiovascular disease and are involved in immune response and inflammatory injury after MI (20, 21). Monocytes/macrophages dominate cellular infiltration during the first 2 weeks after MI and are involved in infarct wound healing (22). In the early stages of MI, injury to the heart causes monocytes to infiltrate the area of infarction and then differentiate into macrophages (23). Macrophages are heterogeneous in their differentiation and function, with M1 macrophages secreting proinflammatory factors and M2 macrophages secreting anti-inflammatory factors (24). Studies have shown that healing after MI involves inflammatory Ly-6C^high^ and reparative Ly-6C^low^ biphasic accumulation of monocytes/macrophages (20, 25). Ly-6C^high^ monocytes are most abundant on day three of MI and decline on day seven, engulfing necrotic and apoptotic cardiomyocytes and reducing the size of the infarct (25, 26). Ly-6C^low^ macrophages promote scar formation, increase interleukin (IL) -21 receptor expression and prevent early infarct expansion (27). Studies have shown that treatment of myocardial infarcted mice with the CXC-motif receptor 4 (CXCR4) blocker AMD3100 significantly reduces the content of neutrophils and Ly-6C^high^ monocytes, which contributes to the acceleration of the inflammatory phase and promotes the healing of myocardial infarcts (28). At 4-7 days of MI, Ly-6C^low^ monocytes are recruited to the infarct region *via* C-X3-C Motif Chemokine Receptor 1(CX3CR1) and promote the repair process (20). C-C Motif Chemokine Receptor 2(CCR2)- and CCR2+ macrophages coordinate monocyte recruitment after myocardial injury (29, 30). Mesenchymal stromal cells (MSCs) have anti-inflammatory effects and cardioprotective functions. In a mouse model of MI, MSCs mediated the transformation from a proinflammatory phenotype to an anti-inflammatory phenotype of macrophages in the infarcted region *via* IL-10, reducing apoptosis of cardiomyocytes and improving cardiac function (31). Treatment of a mouse model of MI with CCR2 antagonists resulted in increased survival of MSCs in the infarcted region and reduced cardiomyocyte death (32). Targeting the recruitment and differentiation of monocytes and macrophages at different stages of MI is an effective strategy for the treatment of MI. DCs can control monocyte/macrophage homeostasis during post-infarction healing, with DCs levels peaking on day 7 of MI. Studies have shown that mice exhibit left ventricular function deterioration and remodeling 7 days after DCs ablation, and MI disrupted by DCs enhances monocyte/macrophage recruitment (33). Decreased DCs numbers and increased macrophage infiltration play a protective role in post-infarction inflammation and subsequent healing, improving cardiac function and preventing adverse cardiac remodeling (34). Interleukin-37 (IL-37), an inhibitor of innate and adaptive immunity, enabled DCs to acquire the characteristics of tolerogenic DCs (tDCs). IL-37 also can induced regulatory T cells, attenuated inflammatory cell infiltration in the infarcted heart, reduced myocardial fibrosis and improved cardiac function, suggesting that modulation of DCs could be a therapeutic strategy for MI (35, 36).

### 2.2 Role of granulocytes in MI

Granulocytes are mainly divided into neutrophils, eosinophils, and basophils (37). Neutrophils include the proinflammatory N1 subgroup and the anti-inflammatory N2 subgroup. The initial MI contained more N1 neutrophils, and N2 expression was elevated during subsequent anti-inflammatory repair (38). Studies have shown that neutrophil deficiency leads to a decrease in Ly-6C^high^ monocytes in mice with macrophage polarization to the M2 phenotype, promoting cardiac repair in MI (39). Reducing neutrophils during inflammation is an effective therapeutic strategy in mice with MI. Blockade of the proinflammatory factor S100A9 secreted by neutrophils reduces the number of neutrophils and monocytes/macrophages, providing an anti-inflammatory environment in the infarcted region and significantly improving cardiac function (40). Studies showed that immature CD10neg neutrophils promote the immune response to inflammation in MI by enhancing Interferon-gamma (IFN-γ) production in CD4 T cells (41). Additional studies have shown that gasdermin D (GSDMD) deficiency in the infarcted heart reduces neutrophil and monocyte content, decreases the extent of MI, and improves cardiac function (42). It is suggested that upstream gene regulation of neutrophils and monocytes is an effective way to treat MI. Eosinophils (EOS) are toxic effector cells that are significantly increased in the blood and heart of myocardial infarcted mice compared to normal mice in the infarcted region. Besides, EOS reduces cardiomyocyte death by secreting substances such as Th2 cytokines, reduces the accumulation of pro-inflammatory cells, enhances neutrophil adhesion, and has a cardioprotective function (43). Additionally, studies have shown that in ST-segment elevation MI in patients and mice, activated eosinophils are recruited to the infarct zone, resulting in a decrease in the number of eosinophils in the blood, attenuating the polarization of anti-inflammatory macrophages and promoting the inflammatory phenotype of MI (44). Crucially, interleukin (IL)-5 secreted by macrophages and CD127+ cells mediate eosinophil development in peripheral blood and infarcted myocardium, promoting recovery from cardiac dysfunction after MI (45). Basophil levels are highest between 3 and 7 days after MI (46). Basophils promote healing and proper scar formation and regulate cardiac remodeling in the late phase of MI by enhancing levels of reparative macrophages and basophil-derived cardiac IL-4 and IL-13 in the infarcted heart (46, 47).

### 2.3 Role of lymphocytes in MI

Specific immune responses are critical for wound healing after MI, and after the onset of MI, a large number of T and B cells are recruited in the area of cardiac injury (17). T cells exert immune functions through lymphatic and blood circulation and are mainly divided into CD4+ T cells, CD4+ T cells are mainly divided into Helper T cells (Th), Regulatory T cells (Tregs), and CD8+ T cells (Tc) (48, 49). The cells have multiple phenotypes and play an immune role by activating other immune cells involved in the regulation of MI (50–53). Tregs cells have an immunosuppressive capacity and are enriched in myocardial infarcted mice, inhibiting the proliferation of CD4 and CD8 T cells and their IFN-γ production, promoting infarct repair (54–56). After MI, T cell activation is driven by recognition of the heart’s antigens, and CD4+ T cells promote the healing of myocardial infarct wounds (57, 58). Studies have shown that CD4+ T cell-specific ablation promotes macrophage polarization and contributes to reducing cardiac fibrosis and increasing cardiomyocyte proliferation in young mice (59). After acute MI in mice, CD8 T lymphocytes are recruited and activated in ischemic heart tissue and release granzyme B, leading to apoptosis, adverse ventricular remodeling, and deterioration of myocardial function (60). Infarcted hearts with infiltration of CD4(+)Foxp3(+)CD73(+) regulatory T cell help prevent adverse ventricular remodeling and improve cardiac function after MI by inhibiting inflammation and directly protecting cardiomyocytes (54, 61). In addition, studies have shown that Treg cells reduce the recruitment of IL-17+γδT cell and increase survival in mice with MI (62). CXCR4 antagonist POL5551 attenuated inflammatory gene expression in monocytes and macrophages by enhancing the action of Treg cells and attenuated left ventricular remodeling and systolic dysfunction, suggesting that enhancing Treg cell expression is important for restoring myocardial function (63, 64).

B cells can influence inflammation and remodeling after MI, and the recruitment of pro-inflammatory monocytes into the heart by mature B lymphocytes leads to increased infarct size and worsening cardiac function and can be a promising target for MI therapy (50). Studies have shown that depletion of mature B lymphocytes in mice with MI effectively inhibits C-C Motif Chemokine Ligand 7(CCL7)production and Ly6Chigh monocyte recruitment, improving cardiac function and treating myocardial injury (65). Rituximab is a monoclonal anti-CD20 antibody targeting human B cells (66). MI patients with peak depletion of B cells by rituximab injection on the sixth day after infarction significantly improved myocardial injury and promoted recovery of cardiac function (67). Regulatory B cells (Bregs) have therapeutic potential in a mouse MI model by reducing CCR2-mediated Ly-6C^high^ monocyte infiltration, inhibiting cardiac recruitment of proinflammatory monocytes, and improving cardiac function (68). Bone marrow B-cell proliferation ceases within 24 hours of MI, and increasing the level of B cells from bone marrow significantly improves cardiac function and reduces infarct size after MI (69). The complexity of the role of lymphocytes in MI brings difficulties to the treatment. It is necessary to accurately detect the time point of MI in order to determine the role of lymphocytes in targeted and effective therapy.

## 3 Animals model of MI

**Figure 2:**
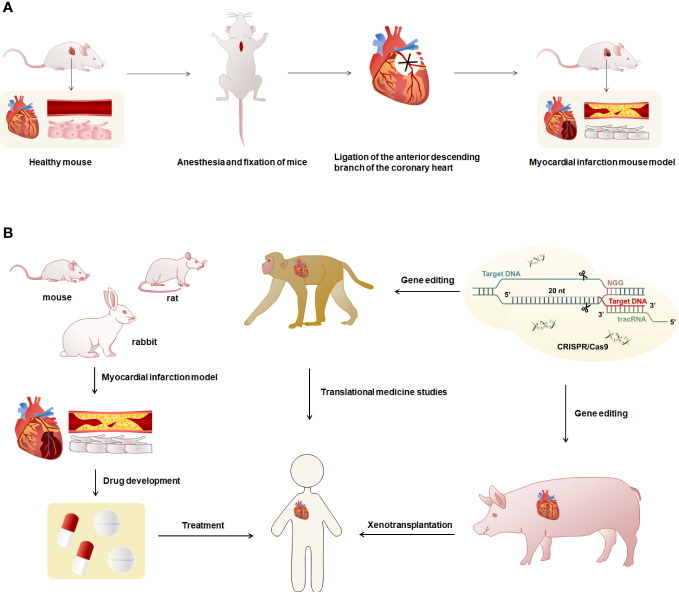
Overview of modeling and uses of animal models of MI. **(A)** The modeling method of the MI model was characterized using mice as an example. First, the mouse was anesthetized and fixed, exposed the heart, and ligated the anterior descending branch of the cardiac coronary artery using sutures to obtain the MI mouse model. Myocardial infarct mice had necrosis of cardiomyocytes and blocked blood vessels. **(B)** Mice, rats and rabbits have small hearts and cannot be used for human heart transplantation. They can be used to prepare models of MI for research such as drug development. The hearts of monkeys and pigs are highly similar to humans, and gene editing techniques can be used to reduce or avoid immune rejection in human organ transplants.

In cardiovascular disease research, animal models are widely used in the exploration of pathogenesis and drug development. The establishment of experimental animal models of MI is important for the in-depth study of the pathology and treatment-related mechanisms of MI (9). The study of animal models that are highly consistent with the phenotype of human MI can provide a more comprehensive understanding of the progression of immune responses and the function of immune cells in MI and contribute to the therapeutic research of MI (70). Currently, the animals commonly used to make models of MI are mice, rats, rabbits, pigs, and monkeys (9, 71–73). Coronary artery ligation is the most commonly used method for modeling MI (**Figure 2A**) (9, 71, 73). The coronary artery stenosis or occlusion caused by ligation leads to ischemia and necrosis of the coronary artery feeding myocardium, which leads to MI in animal models, and produces the same pathological process and immune response as human MI. Real-time monitoring and evaluation of the modeling process through an electrocardiogram, pathology, and serum enzymology can achieve better clinical application (9, 71, 73).

Most studies have used mice for coronary artery ligation to construct animal models of MI (73). Studies have generally used 8-12-week-old female BALB/c mice (74), male C57BL/6 mice (75), and C57BL/6J mice to induce MI (65). Other studies have used 9-13-week-old adult male C57BL/6J mice (76) or female 10-12-week-old NOD-SCID IL2Rgamma (null) mice (31) to construct the MI models. It was shown that unlike male C57BL/6 mice in the same experiment, female MI mice did not exhibit the corresponding EOS deficient phenotype (43). Therefore, it is necessary to distinguish between male and female animals when selecting animals. For rat MI models, studies have generally used 7-10-week-old female Wistar-Kyoto (WKY) rats (70), adult female Wistar rats (72), 5-7-week-old female Sprague-Dawley (SD) rats (77), male SD rats (78), and male SD rats (79). Moreover, most models of MI in rabbits have been performed on New Zealand White rabbits using coronary artery ligation (80). Studies have also been performed in New Zealand White rabbits using thrombogenic coils placed in circumflex arteries to induce closed thoracic MI, describing post-infarction remodeling in small animal models for the first time in which the pericardium remains intact after coronary artery occlusion, providing a more physiologically and clinically consistent in a *vivo* detection system for left ventricular dysfunction after MI (71).

Rodent models have physiological features similar to human cardiac anatomy but are smaller in size (81). The cardiac anatomy and physiology of pigs are more similar to humans, especially the structure, size, and distribution of the coronary arteries, and can accurately mimic the phenotype of human MI (82, 83). In some studies, female and male crossbreed Landrace X Large White pigs were used to induce MI by double ligation of the first marginal branch of the left circumrotation artery 1.5 cm distal to the atrioventricular sulcus after left thoracotomy (84). In addition, studies on adult female Yucatan mini-pigs (70) or Mangalica pigs (85) in which MI was induced by balloon catheter occlusion of the coronary arteries for 90 minutes. There are also studies in which MI models were constructed in female Yorkshire pigs by open-heart surgery and LAD ligation of the distal second diagonal branch (77). Rhesus monkeys are genetically and physiologically similar to humans (86). The distribution of the heart and coronary arteries in rhesus monkeys is highly similar to humans, making them one of the best choices for preparing models of MI (87, 88). Studies generally construct MI models by ligating the left anterior descending coronary artery in 2-3 year old rhesus monkeys (88, 89). One study has used the gene-edited pig heart and xenotransplant it into a baboon, managing the severe immune rejection that occurs with xenotransplants and keeping the baboon alive for more than two years (90). Rodent models of MI can be used for research on biopharmaceuticals, monkeys can be used for research in translational medicine, and pigs have the potential to be used for heart xenotransplantation. Furthermore, the application of gene editing technology to MI mapping and allogeneic organ transplantation in heart can effectively control the occurrence of immune rejection (**Figure 2B**). In the studies of the mechanisms and pathways of MI, the selection of appropriate animals for modeling according to different research purposes and practical situations can help to explore the research methods of MI.

## 4 Biomaterials for MI repair

Cardiac tissue engineering and related biomaterials are emerging tools in the treatment of myocardial infarction (**Supplementary Table 1**) (91, 92). Basic cardiac tissue engineering involves the inoculation of cardiomyocytes or stem cells onto synthetic or natural biocompatible materials *in vitro* and transplantation into infarcted areas of the heart to promote repair of myocardial damage, with the scaffold degrading as the cells integrate with the organism’s tissue (91, 93). As an emerging strategy in tissue engineering, cardiac patches are a focus of research in myocardial tissue repair engineering (94). Cardiac patches are artificial materials that deliver regenerable cells or bioactive molecules to the site of MI for cardiac repair such as myocardial regeneration and can be used as a novel delivery system for cellular therapies and MI repair factors, with almost no immune rejection when implanted *in vivo* (95). Studies have embedded therapeutic synthetic cardiac stromal cells (synCSC) into the decellularized myocardial extracellular matrix (myoECM) to generate an artificial cardiac patch (artCP) with therapeutic characteristics of stem cells. The artCPs significantly reduced fibrosis and infarct size, increased surviving myocardial tissue, and improved cardiac function in rat and pig models of MI, overcoming the limitations of using live stem cells and representing a very promising therapeutic strategy (77). Electrical conduction abnormalities in infarcted myocardium induce adverse myocardial remodeling, causes almost no immune rejection, and limit the action of cardiac regenerative drugs (96). A study has developed a combination of electrospinning of gelatin methacryloyl (GelMA) electrospinning and choline-based bio-ionic liquid (Bio-IL) to construct the cardiac patch, GelMA/Bio-IL, which has a mechanical and electrical conductivity similar to that of native myocardium (97). GelMA/Bio-IL is tightly coupled to mouse myocardium to provide stable mechanical properties to the damaged myocardium and restore electromechanical coupling at the site of MI, reducing cardiac remodeling and maintaining normal function (97). Besides, another study developed an electroactive engineered cardiac patch, silk fibroin, and polypyrrole engineered cardiac patch (SP50 ECP), which significantly expressed cardiac marker proteins with good contractility and electrocoupling properties. SP50 ECP can effectively improve left ventricular remodeling in MI, restore ejection function (EF) and other cardiac functions, promote synchronous contraction of CM in the normal scar area of the myocardium, and effectively reduce the susceptibility to the arrhythmia in the rats with MI (98). During MI episodes, ischemia and hypoxia lead to myocardial cell damage and necrosis, inducing an increase in reactive oxygen species (ROS) that exacerbate tissue damage and cardiac remodeling (99). ROS-responsive biomaterials are considered promising antioxidant candidates for MI therapy, and attenuating oxidative stress in MI is beneficial for reducing inflammation and protecting cardiac tissue. Some studies have designed PFTU/gelatin (PFTU/Gt) fibrous patches with excellent antioxidant activity and ROS-responsive degradability to effectively attenuate oxidative stress in the unfavorable tissue microenvironment *in vivo* after MI in rats, and PFTU/Gt also can inhibit apoptosis, reduce the expression of proinflammatory-related genes, improve cardiac function and angiogenesis, and attenuate poor left ventricular remodeling (100). Currently, microneedle patch is a novel MI treatment that attenuates left ventricle remodeling by mechanical support and is compatible with minimally invasive implantation. Some studies have developed microneedle patches inspired by honeybee venom stings with unidirectional posterior barbs that firmly self-lock on the heart to provide mechanical support to the myocardium of infarcted rats and pigs, significantly reducing wall stress and strain in the infarcted region and maintaining cardiac function and left ventricular morphology (101). Studies of cardiac patches have avoided the harm to the organism from immune rejection, broadened the boundaries of MI therapy, opened up the new direction of subsequent research.

## 5 Discussion

MI has a complex inflammatory response and damage to cardiomyocytes, and immune cells play a very crucial role in the infarcted area. After the onset of MI, monocytes, macrophages, and other cells accumulate in the infarcted region activate and polarize at different times, produce pro- or anti-inflammatory factors, regulate cardiomyocyte proliferation and apoptosis, and influence cardiac remodeling and healing (102). T lymphocytes and B lymphocytes are recruited to the infarcted region after the onset of the nonspecific immune response and participate in the clearance and repair of damaged cells and tissues (59, 65). The anatomical structure of the rodent cardiac model is significantly different from that of the human hearts in size, but the structure of pigs and rhesus monkeys’ hearts is basically the same as that of the human heart. The MI model constructed from pigs and rhesus monkeys can be effectively used for the development of clinical treatment (81–83, 88). With the progress of gene editing technology, studies have already been done to genetically edit pigs to make them resistant to human antibodies and immune cell killing, almost completely avoiding immune rejection from allogeneic organ transplants (90, 103). The pigs breeding cost is far lower than the monkeys, therefore, pig hearts hold the potential for use in allogeneic organ transplants in humans suffering from severe MI. In the development of biomaterials, artificially prepared engineered cardiac patches offer promising new approaches for the treatment and prognosis of MI (101). By optimizing cardiac patch materials loaded with self-proliferating cells or biologic factors with therapeutic benefits, immune rejection is minimized, repair of the infarct site is improved, and integration of the patch with host heart survival and function is promoted, providing damaged regeneration of the heart with mechanical support (95). Targeted modulation of immune cells in MI is a promising strategy, and based on the dual role of many immune cells, there is a need to explore the mechanism of action of immune cells in MI using suitable animal models, and combine bioengineering tools such as cardiac patches to investigate in depth the treatment and repair of human MI.

## 6 Conclusion

In this review, we summarize the recruitment and differentiation of major immune cells in nonspecific and specific immunity in MI and elucidate that immune cells of different typologies have different functions in various periods of MI. Additionally, this review also summarizes the current technologically mature animal models of MI and the potential clinical applications of the emerging cardiac patch in the treatment of MI. The in-depth exploration of immune cells provides effective approaches for the treatment of MI.

## Author contributions

QF, QL, HZ, GG, and DW wrote the manuscript. QF, QL, HZ, LS, CL, YJ, GG, and DW collected the references and prepared figures. All authors contributed to the article and approved the submitted version.

## Supplementary material

Supplementary Figure 1 Immune cells that play a major role in the three phases of myocardial infarction.
