# Supplementary figures and images for: The role of major immune cells in myocardial infarction
Source: Front Immunol. 2023 Jan 19;13:1084460. doi: 10.3389/fimmu.2022.1084460 (PMC9892933; doi:10.3389/fimmu.2022.1084460)

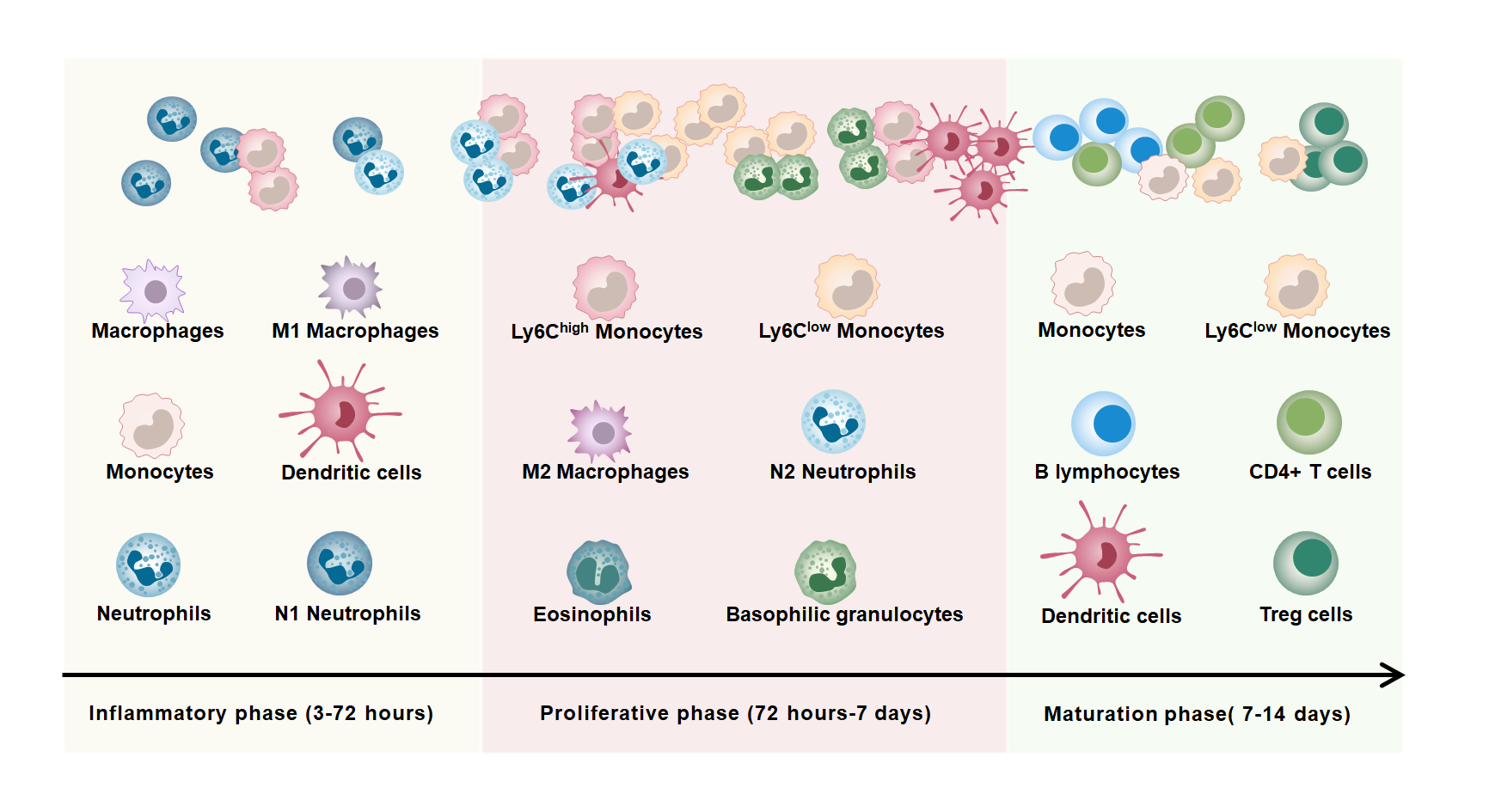

Supplement: Supplementary Figure 1 — Immune cells that play a major role in the three phases of myocardial infarction. [file Image_1.tif]
