# Supplementary material for: The role of major immune cells in myocardial infarction
Source: Front Immunol. 2023 Jan 19;13:1084460. doi: 10.3389/fimmu.2022.1084460 (PMC9892933; doi:10.3389/fimmu.2022.1084460)
Supplement: Supplementary file 2 [file Table_1.docx]

Supplementary Material

# Supplementary Table 1: Engineered tissue patch for the treatment of myocardial infarction.

| Name | Animal model of myocardial infarction | Loaded substances | Treatment effect on myocardial infarction | Regulation of immune rejection | Reference |
| --- | --- | --- | --- | --- | --- |
| Reduced graphene oxide functionalized electrospun silk fibroin (rGO/silk) biomaterials | Rats | - | Restoration of anisotropic electrical microenvironment in the infarcted myocardium improves post-infarct myocardial function. | - | (1) |
| Self-interlocking microneedle patches | Pigs | - | Significantly reduced wall stress and strain at the infarct and maintained the function and morphology of the left ventricle. | - | (2) |
| Electrospun composite polyurethane/gelatin (PFTU/Gt) fibrous patches | Rats | - | Reduced left ventricular remodeling and improved cardiac function. | - | (3) |
| Silk fibroin and polypyrrole engineered cardiac patch (SP50 ECP) | Rats | - | Effectively improves left ventricular remodeling, restores heart function, and improves the propagation of electrical impulses. | - | (4) |
| Mechanically robust multifunctional cardiac patch | Rats | - | Decreased apoptosis and induced angiogenesis. Maintains cardiac function and left ventricular morphology. | Local inflammatory response is suppressed | (5) |
| EVC Spheroids cardiac patch | Nude mice | - | Helps restore heart function after myocardial infarction | - | (6) |
| Fibrin based cardiac patch | Mice | Neuregulin-1 | Reduces apoptosis, decreases the size of myocardial infarction and improves cardiac function. | - | (7) |
| Gelatin methacryloyl hydrogel microneedle patch | Rats | Galunisertib | Improved heart function and reduced cardiac fibrosis. | Does not lead to increased macrophage and neutrophil infiltration and has anti-inflammatory effects | (8) |
| Ti3C2Tx MXene-PEG composite hydrogel | - | Human induced pluripotent stem-cell derived cardiomyocyte | Improved electrophysiological coupling of the patch to the infarcted area | - | (9) |
| Poly (ester carbonate urethane) urea cardiac patch | Rats | Adipose-derived stem cells | Improved left ventricular contractility and remodeling | - | (10) |
| Hybrid Polymer Nanofiber/Collagen Patches | - | Hepatocyte growth factor and insulin-like growth factor | Reduces cell loss due to infarct-induced apoptosis, thereby protecting the remaining functional myocardium | - | (11) |
| ASP-A35m hydrogel cardiac patch | Mice | Human follistatin-like 1 | Reduces fibrosis, inhibits scarring, and enhances heart function | No increase in leukocyte and macrophage infiltration, no additional inflammation | (12) |
| Microneedle-mesenchymal stromal cell-secreted factors-nanoparticles cardiac patch | Rats | Mesenchymal stromal cell-secreted factors | Stimulates cardiac remodeling, accelerates angiogenesis and promotes cardiac repair | Upregulation of anti-inflammatory-related genes and downregulation of pro-inflammatory-related genes | (13) |
| Mechanical-electrical coupling hydrogel patch | Rats | Adipose-derived stem cells | Reduces infarct size, relieves fibrosis, and inhibits ventricular dilation | - | (14) |
| Micropatterned conductive elastomer patch based on poly (glycerol sebacate)-graphene | Rats | - | Reduced infarct size and myocardial fibrosis, and reduced collagen deposition. | Reduces inflammatory response in vivo and has good biocompatibility | (15) |
| Layer-by-layer-human cardiac muscle patches | Mice | - | Improved heart function and reduced infarct size | - | (16) |
| Gold nanoparticle coated cholecystic extracellular matrix | Rats | - | Regulates left ventricular contraction and promotes hemodialysis and restoration of blood flow in the damaged myocardium | - | (17) |
| Collagen and silk-fibroin cardiac and polyaniline patches | Rats | - | Better left ventricular remodeling characteristics shown in infarcted heart models | - | (18) |
| Autologous atrial appendage micrografts with extracellular matrix patch | Pigs | - | Reduces infarct size and attenuates inflammatory response | Inhibition of acute inflammatory reactivity | (19) |
| Engineered biomimetic cardiogel patch | Rats | rat bone marrow stem cells | Restore the function of the heart and enhance the recovery of the heart muscle tissue | - | (20) |
| Self-unfolding graphene oxide-poly (vinyl alcohol) microneedle patch | Mice | Vascular endothelial growth factor | Reduces myocardial fibrosis, increases the number of new blood vessels, and improves myocardial function. | - | (21) |
| PU/PANI/SiO2 electrospinning sub-micron fiber patch | - | - | Simulates myocardial ECM and improves signal transduction. | - | (22) |
| PLLA-HA/rhACE2-electrospun fibrous patch | Mice | - | Reduction of cardiomyocyte apoptosis and inhibition of cardiac fibroblast proliferation | - | (23) |
| Ang-1-hiPSC-aCM seeded patch | Rats | - | Reduce apoptosis, induce angiogenesis, inhibit left ventricular dilation and improve left ventricular systolic function | - | (24) |
| Double-layered adhesive microneedle bandages (DL-AMNBs) | Rats | - | Reduction of adverse left ventricular remodeling after myocardial infarction. | - | (25) |
| POG1 engineered cardiac patch | Rats | - | Reduction of left ventricular remodeling and restoration of cardiac function | - | (26) |
| 3D cardiac mesh (cMesh) | Rats | - | Reduces fibrosis, increases left ventricular thickness and enhances cardiac function. | - | (27) |
| Nitrate-functionalized patch | Rats and pigs | - | Produces mitochondria-targeted cardio protection and enhanced cardiac repair | Significant increase in the number of anti-inflammatory and repair macrophages and decrease in the number of pro-inflammatory macrophages. | (28) |
| Bacterial cellulose membrane patches | Rats | Bone marrow mesenchymal stem cells | Repairing heart damage | - | (29) |
| Upscaled EHT patch | Rabbits | Human induced pluripotent stem cell-derived cardiomyocytes | Improved heart function and reduced the size of scars | The patch itself has a low immune activation rate | (30) |
| Reduced graphene oxide (rGO)/silk patch | Rats | - | Improves heart function and reduces fibrosis of heart tissue. | rGO/silk patch induced infection/immunity and pro-inflammation, rGO has anti-inflammatory effects. | (31) |
| Decellularized placenta Bioengineered cardiac patch | Rats | Human-induced pluripotent stem cell-derived cardiomyocytes | Reduced infarct size and increased cell retention and neovascularization. | - | (32) |
| Perfusable multifunctional epicardial device | Pigs | - | Promotes angiogenesis and allows infiltration of reparative cells, expanding the efficacy of cardiac repair. | Only a few CD3+ lymphocytes were activated. | (33) |
| Biocompatible hydrogels in situ cardiac patch | Pigs | Hydrogels containing induced pluripotent stem cells-derived cardiac progenitor cells or mesenchymal stem cells-derived exosomes | Reduced cardiac remodeling and improved cardiac function after myocardial infarction. | Reduces immune cell infiltration and attenuates immune response | (34) |
| Decellularized porcine myocardial extracellular matrix scaffold and synthetic cardiac stromal cells artificial cardiac patch (artCP) | Pigs and rats | - | Reduce scarring, promote angiogenesis and improve heart function to support cardiac recovery. | ArtCP transplantation causes almost no immune rejection. | (35) |
| ECM/SF patches | Rats | Gold nanoparticles and mesenchymal stem cells | Significant reduction in infarct size, facilitating cardiac regeneration | - | (36) |
| Dopamine-gelatin/ dopamine-functionalized polypyrrole hydrogel patch | Rats | - | Inhibits left ventricular remodeling after myocardial infarction and significantly improves myocardial function. | - | (37) |
| Bionic microvessels- cardiac stromal cell patch | Rats and pigs | - | Promotes heart function and new blood vessel formation | Patches inhibit pro-inflammatory cytokines | (38) |
| Four-dimensional (4D) cardiac patch | Mice | Human mesenchymal stromal cells and human endothelial cells | Enhanced cardiac maturation | - | (39) |
| Conductive Ti2C-cryogel | Rats | Cardiomyocytes | Improves cardiac function, reduces infarct size. | Inhibits the inflammatory response. | (40) |
| hCMPs composed of hiPSC-CMs and nanoparticles that contained CHIR99021 and FGF1 | Mice | Fibroblast growth factor 1 and CHIR99021 | Increased angiogenesis at the infarct border and reduced apoptosis | - | (41) |
| ROS-responsive polyurethane fibrous patches | Rats | Methylprednisolone | Effectively improves the reconstruction of cardiac function and reduces the infarct size. | Anti-inflammatory effect | (42) |
| Epicardial atrial appendage micrograft patch | Mice | - | Rescuing functional heart tissue and limiting fibrosis in myocardial infarction | Modulates the inflammatory response in a manner that promotes functional myocardial recovery. | (43) |
| Gelatin methacryloyl/bio-ionic liquid (GelMA/Bio-IL) patches | Mice | - | Provides mechanical support and restores electromechanical coupling at the site of myocardial infarction, minimizing cardiac remodeling and maintaining normal cardiac function | - | (44) |

# References

1. Zhao G, Feng Y, Xue L, Cui M, Zhang Q, Xu F, et al. Anisotropic Conductive Reduced Graphene Oxide/Silk Matrices Promote Post-Infarction Myocardial Function by Restoring Electrical Integrity. *Acta Biomater* (2022) 139:190-203. Epub 2021/04/10. doi: 10.1016/j.actbio.2021.03.073.

2. Lu Y, Ren T, Zhang H, Jin Q, Shen L, Shan M, et al. A Honeybee Stinger-Inspired Self-Interlocking Microneedle Patch and Its Application in Myocardial Infarction Treatment. *Acta Biomater* (2022). Epub 2022/09/19. doi: 10.1016/j.actbio.2022.09.015.

3. Xie J, Yao Y, Wang S, Fan L, Ding J, Gao Y, et al. Alleviating Oxidative Injury of Myocardial Infarction by a Fibrous Polyurethane Patch with Condensed Ros-Scavenging Backbone Units. *Adv Healthc Mater* (2022) 11(4):e2101855. Epub 2021/11/24. doi: 10.1002/adhm.202101855.

4. Yin Q, Zhu P, Liu W, Gao Z, Zhao L, Wang C, et al. A Conductive Bioengineered Cardiac Patch for Myocardial Infarction Treatment by Improving Tissue Electrical Integrity. *Adv Healthc Mater* (2022):e2201856. Epub 2022/10/14. doi: 10.1002/adhm.202201856.

5. Yao Y, Li A, Wang S, Lu Y, Xie J, Zhang H, et al. Multifunctional Elastomer Cardiac Patches for Preventing Left Ventricle Remodeling after Myocardial Infarction in Vivo. *Biomaterials* (2022) 282:121382. Epub 2022/01/26. doi: 10.1016/j.biomaterials.2022.121382.

6. Liu Y, Zhang Y, Mei T, Cao H, Hu Y, Jia W, et al. Hescs-Derived Early Vascular Cell Spheroids for Cardiac Tissue Vascular Engineering and Myocardial Infarction Treatment. *Adv Sci (Weinh)* (2022) 9(9):e2104299. Epub 2022/01/30. doi: 10.1002/advs.202104299.

7. Chang T, Liu C, Yang H, Lu K, Han Y, Zheng Y, et al. Fibrin-Based Cardiac Patch Containing Neuregulin-1 for Heart Repair after Myocardial Infarction. *Colloids Surf B Biointerfaces* (2022) 220:112936. Epub 2022/10/21. doi: 10.1016/j.colsurfb.2022.112936.

8. Chen H, Fan L, Peng N, Yin Y, Mu D, Wang J, et al. Galunisertib-Loaded Gelatin Methacryloyl Hydrogel Microneedle Patch for Cardiac Repair after Myocardial Infarction. *ACS Appl Mater Interfaces* (2022) 14(36):40491-500. Epub 2022/08/30. doi: 10.1021/acsami.2c05352.

9. Basara G, Saeidi-Javash M, Ren X, Bahcecioglu G, Wyatt BC, Anasori B, et al. Electrically Conductive 3d Printed Ti(3)C(2)T(X) Mxene-Peg Composite Constructs for Cardiac Tissue Engineering. *Acta Biomater* (2022) 139:179-89. Epub 2020/12/23. doi: 10.1016/j.actbio.2020.12.033.

10. Kashiyama N, Kormos RL, Matsumura Y, D'Amore A, Miyagawa S, Sawa Y, et al. Adipose-Derived Stem Cell Sheet under an Elastic Patch Improves Cardiac Function in Rats after Myocardial Infarction. *J Thorac Cardiovasc Surg* (2022) 163(4):e261-e72. Epub 2020/07/09. doi: 10.1016/j.jtcvs.2020.04.150.

11. Kerignard E, Bethry A, Falcoz C, Nottelet B, Pinese C. Design of Hybrid Polymer Nanofiber/Collagen Patches Releasing Igf and Hgf to Promote Cardiac Regeneration. *Pharmaceutics* (2022) 14(9). Epub 2022/09/24. doi: 10.3390/pharmaceutics14091854.

12. Jiang X, Feng T, An B, Ren S, Meng J, Li K, et al. A Bi-Layer Hydrogel Cardiac Patch Made of Recombinant Functional Proteins. *Adv Mater* (2022) 34(19):e2201411. Epub 2022/03/22. doi: 10.1002/adma.202201411.

13. Hu S, Zhu D, Li Z, Cheng K. Detachable Microneedle Patches Deliver Mesenchymal Stromal Cell Factor-Loaded Nanoparticles for Cardiac Repair. *ACS Nano* (2022) 16(10):15935-45. Epub 2022/09/24. doi: 10.1021/acsnano.2c03060.

14. Yu C, Yue Z, Shi M, Jiang L, Chen S, Yao M, et al. An Intrapericardial Injectable Hydrogel Patch for Mechanical-Electrical Coupling with Infarcted Myocardium. *ACS Nano* (2022) 16(10):16234-48. Epub 2022/10/04. doi: 10.1021/acsnano.2c05168.

15. Shi M, Bai L, Xu M, Li Z, Hu T, Hu J, et al. Micropatterned Conductive Elastomer Patch Based on Poly (Glycerol Sebacate)-Graphene for Cardiac Tissue Repair. *Biofabrication* (2022) 14(3). Epub 2022/03/03. doi: 10.1088/1758-5090/ac59f2.

16. Wang L, Zhang J. Layer-by-Layer Fabrication of Thicker and Larger Human Cardiac Muscle Patches for Cardiac Repair in Mice. *Front Cardiovasc Med* (2021) 8:800667. Epub 2022/01/25. doi: 10.3389/fcvm.2021.800667.

17. Nair RS, Sobhan PK, Shenoy SJ, Prabhu MA, Rema AM, Ramachandran S, et al. A Porcine Cholecystic Extracellular Matrix Conductive Scaffold for Cardiac Tissue Repair. *J Biomed Mater Res B Appl Biomater* (2022) 110(9):2039-49. Epub 2022/03/20. doi: 10.1002/jbm.b.35058.

18. Leite FG, Marana JF, de Sá LFT, Alves de Almeida TFR, do Carmo HRP, Chaud MV, et al. Effects of a Collagen Hyaluronic Acid Silk-Fibroin Patch with the Electroconductive Element Polyaniline on Left Ventricular Remodeling in an Infarct Heart Model. *J Biomed Mater Res B Appl Biomater* (2022) 110(7):1651-66. Epub 2022/02/01. doi: 10.1002/jbm.b.35026.

19. Nummi A, Pätilä T, Mulari S, Lampinen M, Nieminen T, Mäyränpää MI, et al. Epicardial Transplantation of Autologous Atrial Appendage Micrografts: Evaluation of Safety and Feasibility in Pigs after Coronary Artery Occlusion. *Scand Cardiovasc J* (2022) 56(1):352-60. Epub 2022/08/26. doi: 10.1080/14017431.2022.2111462.

20. Sharma V, Manhas A, Gupta S, Dikshit M, Jagavelu K, Verma RS. Fabrication, Characterization and in Vivo Assessment of Cardiogel Loaded Chitosan Patch for Myocardial Regeneration. *Int J Biol Macromol* (2022). Epub 2022/10/16. doi: 10.1016/j.ijbiomac.2022.10.079.

21. Fan Z, Wei Y, Yin Z, Huang H, Liao X, Sun L, et al. Near-Infrared Light-Triggered Unfolding Microneedle Patch for Minimally Invasive Treatment of Myocardial Ischemia. *ACS Appl Mater Interfaces* (2021) 13(34):40278-89. Epub 2021/08/24. doi: 10.1021/acsami.1c09658.

22. Feng J, Shi H, Yang X, Xiao S. Self-Adhesion Conductive Sub-Micron Fiber Cardiac Patch from Shape Memory Polymers to Promote Electrical Signal Transduction Function. *ACS Appl Mater Interfaces* (2021) 13(17):19593-602. Epub 2021/04/27. doi: 10.1021/acsami.0c22844.

23. Qiu Z, Zhao J, Huang F, Bao L, Chen Y, Yang K, et al. Myocardial Fibrosis Reversion Via Rhace2-Electrospun Fibrous Patch for Ventricular Remodeling Prevention. *NPJ Regen Med* (2021) 6(1):44. Epub 2021/08/12. doi: 10.1038/s41536-021-00154-y.

24. Tao Z, Loo S, Su L, Tan S, Tee G, Gan SU, et al. Angiopoietin-1 Enhanced Myocyte Mitosis, Engraftment, and the Reparability of Hipsc-Cms for Treatment of Myocardial Infarction. *Cardiovasc Res* (2021) 117(6):1578-91. Epub 2020/07/16. doi: 10.1093/cvr/cvaa215.

25. Lim S, Park TY, Jeon EY, Joo KI, Cha HJ. Double-Layered Adhesive Microneedle Bandage Based on Biofunctionalized Mussel Protein for Cardiac Tissue Regeneration. *Biomaterials* (2021) 278:121171. Epub 2021/10/09. doi: 10.1016/j.biomaterials.2021.121171.

26. Song X, Wang X, Zhang J, Shen S, Yin W, Ye G, et al. A Tunable Self-Healing Ionic Hydrogel with Microscopic Homogeneous Conductivity as a Cardiac Patch for Myocardial Infarction Repair. *Biomaterials* (2021) 273:120811. Epub 2021/04/22. doi: 10.1016/j.biomaterials.2021.120811.

27. Kim KS, Joo HJ, Choi SC, Kim JH, Park CY, Song MH, et al. Transplantation of 3d Bio-Printed Cardiac Mesh Improves Cardiac Function and Vessel Formation Via Angpt1/Tie2 Pathway in Rats with Acute Myocardial Infarction. *Biofabrication* (2021) 13(4). Epub 2021/08/18. doi: 10.1088/1758-5090/ac1e78.

28. Zhu D, Hou J, Qian M, Jin D, Hao T, Pan Y, et al. Nitrate-Functionalized Patch Confers Cardioprotection and Improves Heart Repair after Myocardial Infarction Via Local Nitric Oxide Delivery. *Nat Commun* (2021) 12(1):4501. Epub 2021/07/25. doi: 10.1038/s41467-021-24804-3.

29. Simeoni RB, Mogharbel BF, Francisco JC, Miyague NI, Irioda AC, Souza C, et al. Beneficial Roles of Cellulose Patch-Mediated Cell Therapy in Myocardial Infarction: A Preclinical Study. *Cells* (2021) 10(2). Epub 2021/03/07. doi: 10.3390/cells10020424.

30. Jabbour RJ, Owen TJ, Pandey P, Reinsch M, Wang B, King O, et al. In Vivo Grafting of Large Engineered Heart Tissue Patches for Cardiac Repair. *JCI Insight* (2021) 6(15). Epub 2021/08/10. doi: 10.1172/jci.insight.144068.

31. Feng Y, Zhao G, Xu M, Xing X, Yang L, Ma Y, et al. Rgo/Silk Fibroin-Modified Nanofibrous Patches Prevent Ventricular Remodeling Via Yap/Taz-Tgfβ1/Smads Signaling after Myocardial Infarction in Rats. *Front Cardiovasc Med* (2021) 8:718055. Epub 2021/09/07. doi: 10.3389/fcvm.2021.718055.

32. Jiang Y, Sun SJ, Zhen Z, Wei R, Zhang N, Liao SY, et al. Myocardial Repair of Bioengineered Cardiac Patches with Decellularized Placental Scaffold and Human-Induced Pluripotent Stem Cells in a Rat Model of Myocardial Infarction. *Stem Cell Res Ther* (2021) 12(1):13. Epub 2021/01/09. doi: 10.1186/s13287-020-02066-y.

33. Huang S, Lei D, Yang Q, Yang Y, Jiang C, Shi H, et al. A Perfusable, Multifunctional Epicardial Device Improves Cardiac Function and Tissue Repair. *Nat Med* (2021) 27(3):480-90. Epub 2021/03/17. doi: 10.1038/s41591-021-01279-9.

34. Zhu D, Li Z, Huang K, Caranasos TG, Rossi JS, Cheng K. Minimally Invasive Delivery of Therapeutic Agents by Hydrogel Injection into the Pericardial Cavity for Cardiac Repair. *Nat Commun* (2021) 12(1):1412. Epub 2021/03/05. doi: 10.1038/s41467-021-21682-7.

35. Huang K, Ozpinar EW, Su T, Tang J, Shen D, Qiao L, et al. An Off-the-Shelf Artificial Cardiac Patch Improves Cardiac Repair after Myocardial Infarction in Rats and Pigs. *Sci Transl Med* (2020) 12(538). Epub 2020/04/10. doi: 10.1126/scitranslmed.aat9683.

36. Dong Y, Hong M, Dai R, Wu H, Zhu P. Engineered Bioactive Nanoparticles Incorporated Biofunctionalized Ecm/Silk Proteins Based Cardiac Patches Combined with Mscs for the Repair of Myocardial Infarction: In Vitro and in Vivo Evaluations. *Sci Total Environ* (2020) 707:135976. Epub 2019/12/23. doi: 10.1016/j.scitotenv.2019.135976.

37. Wu T, Cui C, Huang Y, Liu Y, Fan C, Han X, et al. Coadministration of an Adhesive Conductive Hydrogel Patch and an Injectable Hydrogel to Treat Myocardial Infarction. *ACS Appl Mater Interfaces* (2020) 12(2):2039-48. Epub 2019/12/21. doi: 10.1021/acsami.9b17907.

38. Su T, Huang K, Mathews KG, Scharf VF, Hu S, Li Z, et al. Cardiac Stromal Cell Patch Integrated with Engineered Microvessels Improves Recovery from Myocardial Infarction in Rats and Pigs. *ACS Biomater Sci Eng* (2020) 6(11):6309-20. Epub 2021/01/16. doi: 10.1021/acsbiomaterials.0c00942.

39. Cui H, Liu C, Esworthy T, Huang Y, Yu ZX, Zhou X, et al. 4d Physiologically Adaptable Cardiac Patch: A 4-Month in Vivo Study for the Treatment of Myocardial Infarction. *Sci Adv* (2020) 6(26):eabb5067. Epub 2020/07/09. doi: 10.1126/sciadv.abb5067.

40. Ye G, Wen Z, Wen F, Song X, Wang L, Li C, et al. Mussel-Inspired Conductive Ti(2)C-Cryogel Promotes Functional Maturation of Cardiomyocytes and Enhances Repair of Myocardial Infarction. *Theranostics* (2020) 10(5):2047-66. Epub 2020/02/28. doi: 10.7150/thno.38876.

41. Fan C, Tang Y, Zhao M, Lou X, Pretorius D, Menasche P, et al. Chir99021 and Fibroblast Growth Factor 1 Enhance the Regenerative Potency of Human Cardiac Muscle Patch after Myocardial Infarction in Mice. *J Mol Cell Cardiol* (2020) 141:1-10. Epub 2020/03/15. doi: 10.1016/j.yjmcc.2020.03.003.

42. Yao Y, Ding J, Wang Z, Zhang H, Xie J, Wang Y, et al. Ros-Responsive Polyurethane Fibrous Patches Loaded with Methylprednisolone (Mp) for Restoring Structures and Functions of Infarcted Myocardium in Vivo. *Biomaterials* (2020) 232:119726. Epub 2020/01/07. doi: 10.1016/j.biomaterials.2019.119726.

43. Xie Y, Lampinen M, Takala J, Sikorski V, Soliymani R, Tarkia M, et al. Epicardial Transplantation of Atrial Appendage Micrograft Patch Salvages Myocardium after Infarction. *J Heart Lung Transplant* (2020) 39(7):707-18. Epub 2020/04/27. doi: 10.1016/j.healun.2020.03.023.

44. Walker BW, Lara RP, Yu CH, Sani ES, Kimball W, Joyce S, et al. Engineering a Naturally-Derived Adhesive and Conductive Cardiopatch. *Biomaterials* (2019) 207:89-101. Epub 2019/04/10. doi: 10.1016/j.biomaterials.2019.03.015.
